# Statin therapy improves outcomes in infective endocarditis: evidence from a meta-analysis

**DOI:** 10.1186/s43044-024-00495-1

**Published:** 2024-06-07

**Authors:** Mojtaba Hedayat Yaghoobi, Ehsan Heidari, Arman Shafiee, Niloofar Seighali, Mohammad Reza Maghsoodi, Mahmood Bakhtiyari

**Affiliations:** 1 School of Medicine, Alborz University of Medical Sciences, Karaj, Iran; 2 School of Pharmacy, Shahid Beheshti University of Medical Sciences, Tehran, Iran

**Keywords:** Statin, Infective endocarditis, Meta-analysis, Infectious Endocarditis

## Abstract

**Background:**

Beyond its ability to decrease cholesterol, statin medication has been proved to have a variety of pleiotropic effects, such as anti-inflammatory and immunomodulatory effects. Statins are an appealing therapeutic option for individuals with infective endocarditis because of these effects, as the condition is linked to a strong inflammatory response.

**Methods:**

A comprehensive search was done in Medline/PubMed, Cochrane database (CENTRAL), and Google Scholar to identify relevant studies reporting outcomes of interest (rate of mortality, intensive care unit admission, and embolic events) comparing those who are on statin therapy to nonusers were included. We performed a random effect meta-analysis to pool each study's individual results.

**Results:**

Three articles were included in the study. The pooled results regarding our primary endpoint showed there was a significant reduction in mortality among statin users in all time points (1-year mortality: OR 0.69, 95% CI 0.61–0.79, *I*2: 0%; Chi2 = 0.01; *p* < 0.0001). Meta-analysis for the secondary outcome showed statin users are less frequently admitted to the intensive care unit (OR 0.73, 95% CI 0.59–0.90, *I*2: 0%; Chi2 = 0.00; *p* = 0.0004). The rate of mortality was significantly lower for those with a previous history of cerebrovascular disease who were on statin therapy compared to those without cerebrovascular diseases (CVD).

**Conclusions:**

The results of the present study support a significant association with statin therapy as a potential treatment proposed for individuals at risk of infective endocarditis.

## Background

Infectious endocarditis (IE) is a heart valves affecting infection that causes vegetations with increasing the risk of abscesses, embolization, and fistulas and destructive effects on the tissue [1]. Recent investigations showed the burden of infective endocarditis patients is increasing with an annual incidence of 15/100,000 which causes a mortality rate of 30% in 330 days among the infected patients [2, 3]. The immense burden of this disease could be accounted for by the cumulative use of interventional strategies and intracardiac devices [4]. There are several organisms responsible for causing this situation including but not limited to Staphylococcus aureus, streptococci, and enterococci groups [5]. Viridans streptococci (oral streptococci) is the 2nd cause after Staphylococcus aureus known for IE [6]. Congenital or acquired cardiac defects and episodes of short bacteremia are known as risk factors. Clinical trials confirmed the role of antibiotic prophylaxis for infective endocarditis in high-risk patients before invasive dental procedures to prevent from bacteremia caused by dental procedures [7, 8]. The prognosis differs with factors such as the valves affected, associated comorbidities, the pathogen, and surgery necessity [9]. Dangerous consequences following IE such as embolic events highlight the importance of investigating new treatment options, especially those which are routinely consumed among patients with cardiovascular diseases. The anti-inflammatory and antithrombotic effects of statin have brought attention to the repurposed use of this drug for preventing adverse outcomes among IE patients [3].

There is a lack of meta-analysis on the effect of statin on infective endocarditis and the risk of mortality, ICU admission, and embolic events. Hence, we aimed to provide a systematic review and meta-analysis to address the paucity of evidence by aggregating the published data on this topic in a manner of conducting a meta-analysis.

## Methods

Our meta-analysis was conducted following the PRISMA statement's recommendations [10].

### Literature search and eligibility criteria

We searched Medline/PubMed, Cochrane database (CENTRAL), and Google Scholar up to February 5, 2023, for relevant studies that addressed our study question (PICO; P: All patients with IE; I: statin users; C: non-statin users; and O: evaluating the comorbid outcomes). Review articles, case reports/series, and conference abstracts were excluded. The following search query was used to retrieve the relevant studies: (“HMG CoA Reductase Inhibitor” OR “Statin” OR “HMG-CoA Statins”) AND (“Endocarditis” OR “Infective Endocarditis”). Initially, two authors independently reviewed the papers' titles and abstracts. It was followed by a full-text screening.

### Selection and outcomes

Our primary endpoint was the risk of 1-year mortality. The secondary outcomes were intensive care unit (ICU) admission and embolic events. Furthermore, we performed a comprehensive set of subgroup analyses for our primary endpoint to increase the validity of our findings. All original articles comparing previous statin users to those who had not used statin which reported outcomes of interest were eligible to be included.

### Quality assessment and data extraction

For quality assessments, we used the Newcastle–Ottawa Quality Assessment Form for Cohort Studies [11]. The following data were extracted from the included studies: author, year, country, type of study, population, total patients, age, duration of the cohort, main findings, confounding factors adjusted in the analyzed model, and quality. Quality assessment was done independently by two authors.

### Data synthesis

We performed a random effect meta-analysis for pooling the individual results of each study. Odds ratios with a 95% confidence interval (CI) were used. We used adjusted data in our meta-analysis whenever a study balances its effect sizes for covariates. We performed a comprehensive set of subgroup analyses of several moderators including age, sex, previous history of cardiovascular disease, and previous history of cardiovascular surgery for our primary endpoint to increase the validity of our findings. All statistical analyses were performed in RevMan 5.4. Publication bias was not assessed as there were less than 10 effect sizes in our meta-analysis.

## Results

### Literature search

**Fig. 1:**
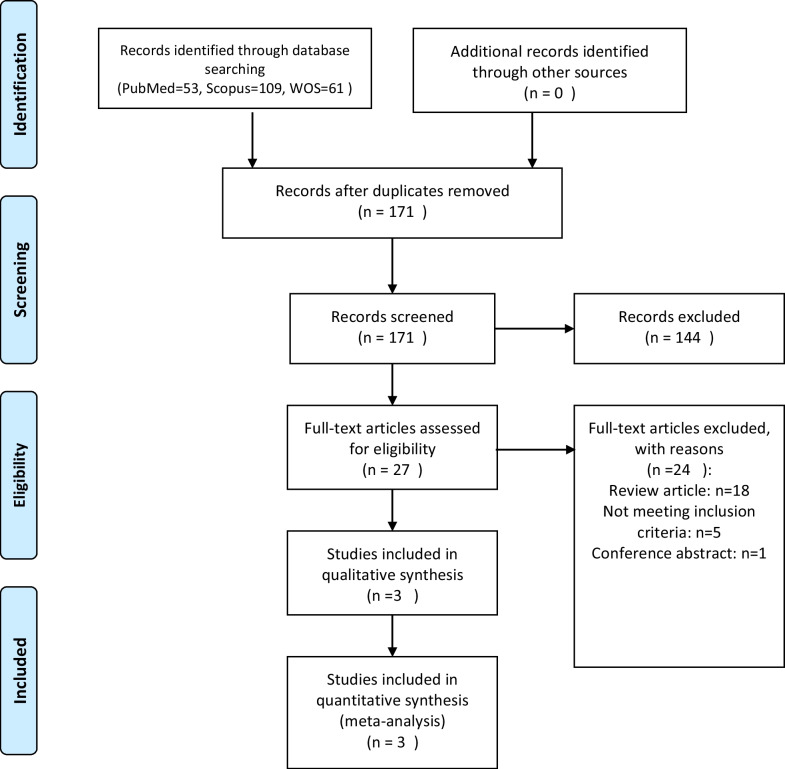
PRISMA flow diagram

Our initial search yielded a total of 223 studies from Medline/PubMed, Cochrane database (CENTRAL), and Google Scholar. After removing duplicates, 171 studies remained (Fig. 1). The titles and abstracts of these studies were screened, and 27 studies were selected for full-text screening. Ultimately, 4 studies met our inclusion criteria and were included in the final analysis.

### Characteristics

Three studies were included [12–14]. Two were conducted in Asia, and one was conducted in America. One article was published recently in 2023, one was published in 2019 and the first study was conducted in 2011. All studies were observational regarding study design. Among the included studies, three studies used a propensity score-matched analysis [12–14]. The detailed characteristic of each study is available in Table 1

**Table 1:** Baseline characteristics of the patients and studies included

ID	Author	Year	Country	Type of study	Population	Total patients	Age	Duration of cohort	Main findings	Confounding factors adjusted in the analyzed model
1	Yu, Si-Yeung [14]	2023	Hong Kong	Retrospective propensity score-matched study	All patients aged 18 years or older with newly diagnosed infective endocarditis (IE) who attended any hospitals	6700	58.0 (18.9)	January 1, 1996, and December 31, 2019	Preadmission statin users had a 31% lower risk of 1-year mortality compared to nonusers, with a hazard ratio (HR) of 0.69 and a 95% confidence interval (CI) of 0.58–0.82 Patients who continued using statins in hospital had a 48% lower risk of 1-year mortality compared to those who discontinued statin use, with an HR of 0.52 and a 95% CI of 0.34–0.78	Age, sex, socioeconomic status, cerebrovascular diseases, chronic kidney diseases, congenital heart diseases, diabetes mellitus, dysrhythmias, drug history, heart failures, implanted cardiac devices, use of prosthetic valves, and myocardial infarction
2	Yang [13]	2014	Taiwan	Retrospective propensity score-matched study	All patients who had hospitalization with the diagnosis of IE	13,584	Statin users: 64.9 (12.7)/non-statin users: 56.6 (19.2)	January 2000 and December 2010	Statin users had a significantly lower risk of in-hospital mortality compared with nonusers, with an adjusted hazard ratio (aHR) of 0.65 and a 95% confidence interval (CI) of 0.49–0.86 The reduction in mortality was sustained over time, showing significant decreases at follow-up intervals: 3 months: aHR 0.68, 95% CI 0.53–0.88 6 months: aHR 0.73, 95% CI 0.58–0.91 12 months: aHR 0.68, 95% CI 0.55–0.84 Statin therapy was also associated with a reduced risk of: Admission to intensive care units (ICU) Shock events The need for mechanical ventilation However, statin use did not significantly affect the need for heart valvular replacement surgery	Adjustments were made for clinically relevant variables and for those that showed a statistically significant difference between the two groups at baseline
3	Anavekar [12]	2011	USA	Retrospective propensity score-matched study	Adult patients with a diagnosis of IE who presented to Mayo Clinic (Rochester, MN)	283	63.5 (0.1–98.4)	January 1, 2003–December 31, 2006	After adjusting for the propensity to treat with statins, the benefit attributable to statin therapy was significant, with an odds ratio of 0.30 and a 95% confidence interval of 0.14–0.62; the result was statistically significant with a *p*-value of 0.001 However, no significant difference was observed in the propensity-adjusted rate of 6-month mortality between patients who had and had not undergone prior statin therapy, with a *p*-value of 0.87	Age, male sex Charlson Comorbidity Index, coronary artery disease, myocardial infarction, peripheral vascular disease, hypertension, high cholesterol, diabetes mellitus, thrombotic states, anticoagulation

### Quantitative synthesis

**Fig. 2:**
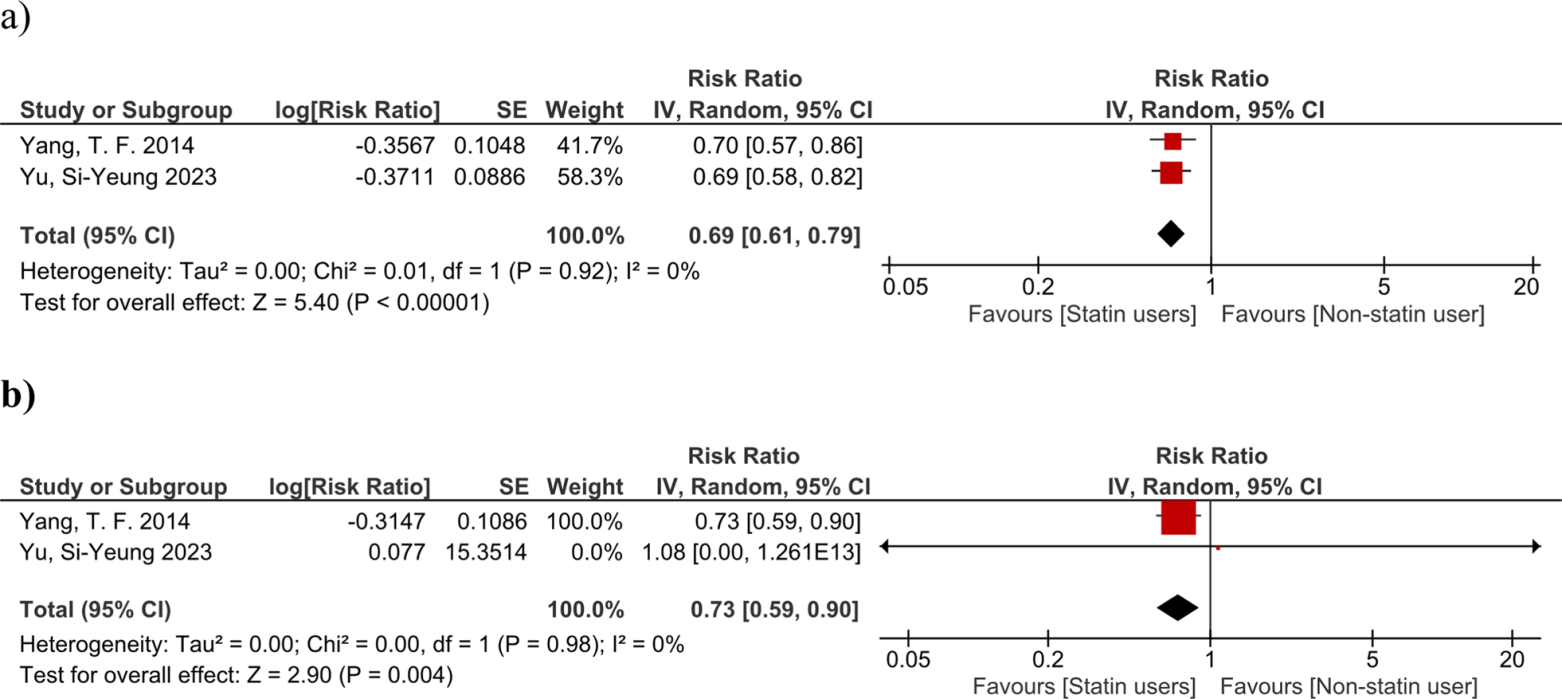
Forest plot showing the results of primary and secondary endpoints: **A** 6-month mortality; and **B** ICU admission

**Fig. 3:**
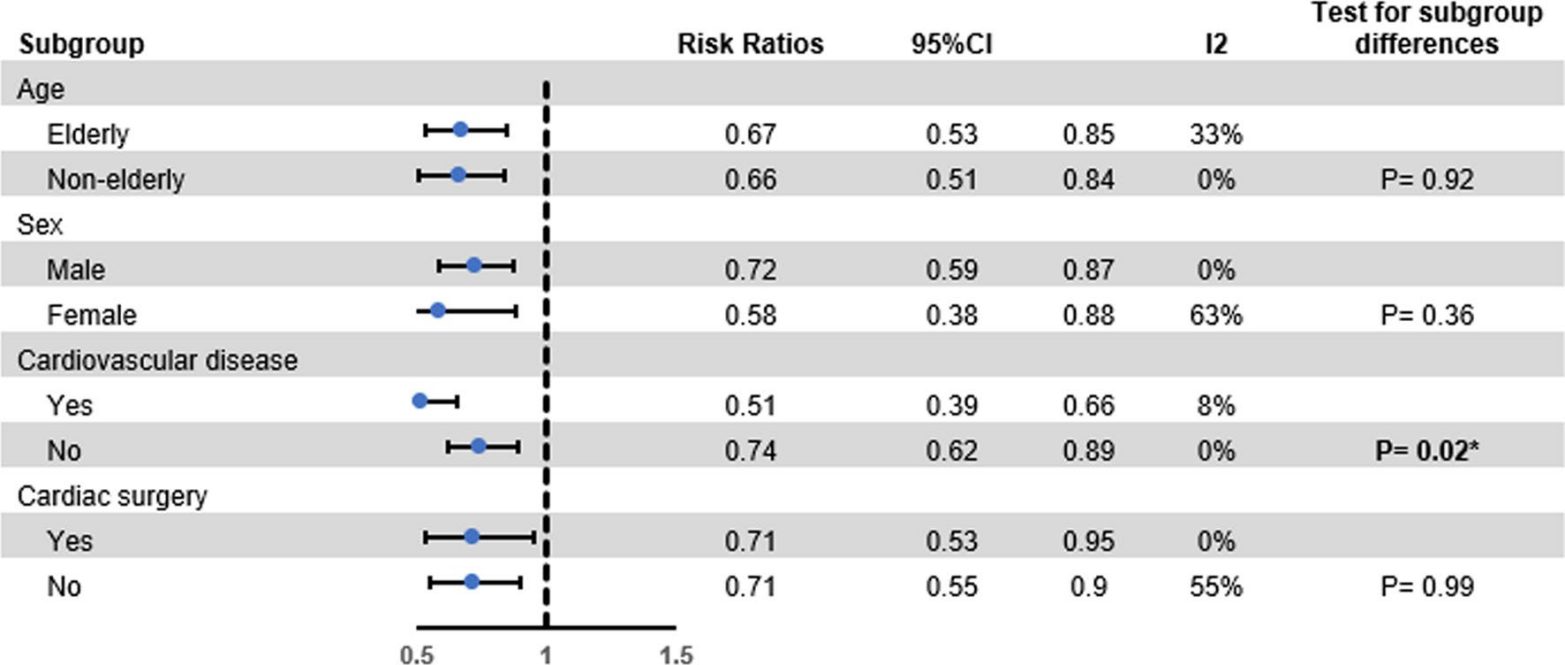
Results of subgroup meta-analysis

The pooled results regarding our primary endpoint showed there was a significant reduction in mortality among statin users in all time points (1-year mortality: OR 0.69, 95% CI 0.61–0.79, *I*2: 0%; Chi2 = 0.01; *p* < 0.0001). Meta-analysis for the secondary outcome showed statin users are less frequently admitted to the intensive care unit (OR 0.73, 95% CI 0.59–0.90, *I*2: 0%; Chi2 = 0.00; *p* = 0.0004) (Fig. 2). A significant decrease in the mortality rate of statin users was found in all our subgroup analyses (Fig. 3). Regarding embolic events, there was not adequate information to perform a meta-analysis. The results of both studies reporting on this outcome showed no significant differences between the groups regarding the rate of embolic events.

### Risk of bias assessment

The results of our quality assessment showed all journal articles were of good quality (Table 2).

**Table 2:** Quality assessment (NOS)

Author	1. Representativeness of the exposed cohort	2. Selection of the non-exposed cohort	3. Ascertainment of exposure	4. Demonstration that outcome of interest was not present at start of study	5. Comparability	6. Assessment of outcome	7. Was follow-up long enough for outcomes to occur	8. Adequacy of follow-up of cohorts	Total
Yu, Si-Yeung	1	1	1	1	2	1	1	1	9
Yang, T. F	1	1	1	1	1	1	1	1	8
Anavekar, N. S	1	0	0	0	1	0	1	0	3

## Discussion

Infective endocarditis is a serious condition that can lead to significant morbidity and mortality. Despite advances in medical and surgical treatment, the mortality rate remains high, particularly in patients with certain comorbidities. Therefore, there is a need for novel therapeutic strategies to improve outcomes in these patients. Statin therapy has been shown to have numerous pleiotropic effects beyond its lipid-lowering properties, including anti-inflammatory and immunomodulatory effects. These effects make statins an attractive therapeutic option for patients with infective endocarditis, as the condition is associated with a significant inflammatory response. The results of our study validate and extend the findings reported by Yu et al. [14] with regard to the efficacy of statin therapy on the clinical outcomes of patients diagnosed with IE. Here, we found that the rate of all-cause mortality was significantly decreased at all time points among those who used statins. Furthermore, ICU admissions also decreased among statin users, which is in contrast to those reported by Yu et al. [14]. The results of our subgroup analysis were based on pooling the findings of two large propensity score-matched cohorts [13, 14]. Interestingly, although Yang et al. [13] reported there was no significant effect of statin therapy for reducing mortality in male patients, the results of our subgroup analysis showed the significant effect of this drug in both sexes. This finding is of high importance since the disease is more prevalent among male patients [3]. Similar findings were observed after performing subgroup analyses for age and previous history of cardiac surgery, which adds to the existing evidence regarding different subgroups of IE.

The exact mechanism by which statins exert their beneficial effects in patients with infective endocarditis is not fully understood. It has been suggested that statins may have an anti-inflammatory effect by inhibiting the production of pro-inflammatory cytokines and chemokines. Additionally, statins may have an immunomodulatory effect by enhancing the phagocytic activity of macrophages and neutrophils. In addition to the anti-inflammatory, antiplatelet, and immunomodulatory effects of statin, the ratio for possible effect of using statins for therapeutic purposes in IE patients may lie behind its potential impact in increasing high-density lipoprotein (HDL-C) levels, which has reported to be decreased in patients with severe infectious diseases [15–17]. In a recent study, low HDL-C was proposed as a possible predictor for complications, in-hospital mortality, and 90-day mortality [15]. Therefore, this causal link may be to account for the potential positive benefits of statins in IE, especially in patients with cardiovascular diseases, as our results showed a significantly lower rate of mortality for those who had a previous CVD and were on statin therapy (*p* = 0.02).

Our study has several strengths that need to be mentioned. First, this is the first meta-analysis of the available evidence regarding statin therapy in IE patients. Conducting a meta-analysis to aggregate the previously published data is highly needed, especially when there is a paucity of information. Second, most of the studies included were propensity score-matched observational studies and we used the adjusted data for our analyses. Propensity score-matched studies are the best alternatives for randomized control trials (RCTs) whenever conducting RCTs is difficult both logistically and financially as we have on this topic. The main limitation of this meta-analysis is its few included studies, resulting in the unavailability of adequate data for performing a meta-analysis for the rate of embolic events. Thus, future research is mandatory in this field to increase the certainty of evidence regarding the efficacy of statin therapy in IE patients. Additionally, the optimal dosing and duration of statin therapy in patients with infective endocarditis are not well established.

## Conclusions

In conclusion, our systematic review and meta-analysis suggest that the use of statins in patients with infective endocarditis is associated with a lower risk of mortality and ICU admission compared to non-statin users. Further studies are needed to confirm these findings and to determine the optimal dosing and duration of statin therapy in this patient population.

## Data Availability

All data have been presented in the manuscript.
